# African American and African-Born Black Women’s Perspectives and Experiences with a Cervical Health Education and HPV-Self Sampling Intervention

**DOI:** 10.3390/healthcare13192389

**Published:** 2025-09-23

**Authors:** Shania Jones, Abubakari Wuni, Adaeze Aroh, Adebola Adegboyega

**Affiliations:** 1 College of Medicine, University of Kentucky, Lexington, KY 40506, USA; shania.jones@uky.edu; 2 Markey Cancer Centre, University of Kentucky, Lexington, KY 40506, USA; 3 College of Nursing, University of Kentucky, Lexington, KY 40506, USA; awu229@uky.edu; 4 Department of Public Health Sciences, Slippery Rock University, Slippery Rock, PA 16057, USA; adaeze.aroh@sru.edu

**Keywords:** cervical cancer screening, HPV self-sampling, health education, African American and African-born women

## Abstract

**Background/Objectives**: A combination of cervical cancer prevention education and the provision of HPV self-collection kits has been found to increase the uptake of HPV testing among women. However, there is limited research evaluating the perspectives and experiences of women who have participated in a cancer prevention education and received a complimentary HPV self-collection kit. We report the experiences of women who took part in Health is Wealth: a cervical health intervention and received a complimentary HPV self-sampling kit for cervical cancer screening. **Methods**: This pilot qualitative study enrolled twenty-four women who participated in one-on-one semi-structured interviews to provide feedback and recommendations for improving future iterations of the intervention. **Results**: Overall, themes related to women’s experiences include empowerment and connections; enlightenment; and accessibility and engagement. In addition, themes related to HPV self-collection include, not as difficult as I thought; convenience; and fear. Our findings suggest that a tailored intervention, which delivers cervical cancer education alongside complementary HPV self-sampling kits while addressing unique barriers experienced by minoritized groups, was well received by African American and African-born Black women. **Conclusions**: The study demonstrates that a culturally adapted intervention combining cervical cancer education with HPV self-sampling kits was positively received by African American and African-born Black women. This emphasizes the interventions’ potential to improve screening uptake by addressing unique barriers and promoting empowerment, convenience, and accessibility.

## 1. Introduction

Cervical cancer is one of the most preventable cancers. Despite significant progress in the prevention, early detection, diagnosis, and treatment of cervical cancer in the U.S., disparities in unequal screening rates and late-stage diagnosis continue to exist among certain groups [1]. Awareness of the benefits of cervical cancer screening, diagnostic methods, and the importance of early follow-up and treatment are among the main factors that encourage women to perform cervical cancer screening [2,3]. Tailored interventions that address the unique barriers experienced by low-resourced and underserved populations like African-born Black women have the potential to mitigate screening barriers and reduce the high burden that cervical cancer places on these populations [4]. African American and African-born black women experience higher rates of cervical cancer incidence and mortality compared to their white counterparts [5]. Notwithstanding shared and racial identity, these two groups have distinct cultural, historical, and healthcare experiences influencing their cervical cancer screening [6,7]. Research that can inform effective approaches to promote cervical cancer screening among populations that experience disparities is critical to improve cervical cancer screening rates.

HPV self-collection was recently approved by the U.S. Food and Drug Administration as a method for cervical cancer screening [8]. HPV self-collection is very relevant for under-screened and unscreened women. This screening modality should be directed at women who have limited access to healthcare. HPV self-collection can circumvent screening barriers like embarrassment, lack of privacy, and discomfort, which may have precluded women from completing conventional clinician-collected cervical cancer screening approaches [9]. HPV self-collection is when a patient uses an HPV collection kit and is provided instructions to collect a cervicovaginal sample [10]. Collected samples are mailed to a laboratory to be tested for high-risk HPV DNA.

Studies suggest that a combination of educational intervention and HPV self-collection can help decrease national racial health disparities in cervical cancer rates [11,12,13]. Given the disparities in cervical cancer outcomes among African American and African-born Black women, and the potential benefits of HPV self-collection to promote cervical screening, we implemented Health is Wealth: a cervical health intervention, which was created for African American and African-born-Black women. This current study describes the experiences, perspectives, and recommendations from the women who participated in the cervical health intervention.

## 2. Materials and Methods

All study procedures were approved by the Institutional Review Board (60704) prior to study commencement. Confidentiality of records and personal information was maintained. Each participant provided informed consent prior to participation in any research activities. The reporting of this qualitative study followed the Standards for Reporting Qualitative Research (SRQR)* created by O’Brien and colleagues [14].

### 2.1. Study Design

The pilot qualitative study employed a descriptive phenomenology approach [15]. Semi-structured individual interviews were conducted to capture the experiences and perspectives of African American and African-born Black women who participated in a cervical health intervention in central Kentucky.

### 2.2. Health Is Wealth: A Cervical Health Intervention

Detailed information about Health is Wealth: a cervical health intervention’s adaptation and usability has been previously published [16]. In brief, the cervical health intervention included the provision of HPV self-sampling kits and a one-time educational session (~75 min) presented by a trained peer health advisor. The intervention was designed to increase cervical cancer screening uptake among African American and African-born Black women via HPV self-collection. During each session, a trained facilitator gave a PowerPoint presentation focused on cervical cancer information, risk factors, common myths about cervical cancer screening, HPV self-collection, and skills to increase self-efficacy to overcome barriers. A case study specific to either African American or African-born Black women was also incorporated. Lastly, participants were provided with resources for facilities that offer free and low-cost cervical cancer screening resources in their community. Health is Wealth: a cervical health intervention was tested in a prospective one-arm quasi-experimental pilot trial among 30 African American and 30 African-born Black women aged 30 to 65 years who were out of compliance with cervical cancer screening. Participants completed pre–post surveys and a 6-month follow-up survey. The eligibility criteria for participation in Health is Wealth: a cervical health intervention were (1) a self-report of not having a Pap smear within the past 3 years, a Pap smear/HPV co-test within the past 5 years, or a HPV test within the past 5 years (per United States Preventive Services Task Force (USPSTF) guidelines); (2) 30–65 years of age, as HPV testing is recommended for women in this age range per USPSTF guidelines; (3) English speaking; (4) self-identify as an African American or African-born Black woman; and (5) have a cell phone to participate in a virtual session. Exclusion criteria for Health is Wealth included current pregnancy, history of hysterectomy, and history of cervical cancer.

### 2.3. Study Population and Recruitment

African American and African-born Black women were recruited by trained Black female research assistants. Approved study flyers were posted in public places throughout the community and on social media. Trained research assistants conducted active recruitment by visiting community settings with high Black clientele, e.g., churches, community centers, and beauty salons. Additional recruitment was completed through snowballing and word of mouth.

All women who participated in the Health is Wealth: a cervical health intervention were invited to participate in a follow-up qualitative interview. Participants were invited via email and or follow-up call six months after participating in the intervention. Eligibility for participation in the follow-up interview included the following: (1) Completion of Health is Wealth: a cervical education session; (2) Completion of pre–post surveys; and (3) willingness to participate in a virtual interview.

### 2.4. Procedure and Data Collection

All interviews were completed between June 2023 and September 2024. Interview meetings were scheduled at the convenience of the participants and conducted via secured Zoom. Interviews were guided with a semi-structured interview guide (See Supplementary Materials for interview guide) developed to collect feedback about the women’s experiences participating in the cervical health intervention and to collect suggestions on improvements for future iterations of the intervention. Interview length ranged from an average of 30 min, and participants were compensated with a $20 electronic gift card for their participation. Interviews were conducted until saturation was reached, meaning we observed that no new insights were emerging from additional interviews. This method ensured that the sample size was sufficient enough to capture the breadth and depth of perspectives on the phenomenon [17,18].

The interviews were conducted in English by a Black female researcher trained in qualitative interview methods. Each interview began with a brief introduction and description of the interview’s purpose. To maintain confidentiality, participants were advised to select a pseudonym to use in the course of the interview. The first question posed was, “as a participant, could you describe your experience participating in the Cervical Health Intervention?”. Participants were then asked additional questions relevant to their participation, such as what they liked or did not like about the intervention; any concerns about the intervention and intervention materials; communication preferences for future iterations; and if they felt additional information should be added to the educational session. The remainder of the guide included questions about HPV self-collection completion. Each interview ended with suggestions or questions the participants may have for the research team. This approach fostered comprehensive discussions and elicited rich qualitative data. Depending on the responses, further probing questions were asked. Data saturation was achieved after 24 interviews, as no information or insights emerged from data collection.

### 2.5. Data Analysis

All 24 digitally recorded interview audio files were transcribed verbatim using Otter.Ai software version 3.87.2. One of the authors listened to the digital audio files while following each transcription to verify transcription accuracy. Transcripts were corrected as needed when any transcription error was detected. We ensured that there were no identifiers in the transcripts. The data analysis was completed manually using a conventional approach without predetermined categorization structures. This process consisted of three stages: preparation, organization, and report writing. We used Microsoft Word to organize the data. We did not use any qualitative data analysis software. During the preparation phase, each interview was used as a unit of analysis. Transcripts were iteratively examined to identify broad categories. To begin the analysis, the researchers used open coding to identify initial concepts and patterns. Axial coding was used to categorize and relate codes to one another, permitting the creation of themes. During the organization phase, units of meaning from each interview were identified, simplified, and explicitly coded. Similar codes were then classified as subcategories and overarching primary categories [19]. Finally, in the reporting phase, the latent meaning of the data was presented as the study’s findings. The credibility of the findings was further bolstered by meticulous attention to the content analysis process. This included selecting meaningful units, categorizing data, and recognizing similarities and differences among categories. Additionally, a complete audit trail was established to allow other researchers to verify the techniques used in this study [20].

## 3. Results

Participants’ (n = 24) demographic characteristics are represented in Table 1. Our sample included ten African Americans (41.7%) and fourteen African-born Black women (58.3%). The majority of the African-born Black women originated from Cameroon and Nigeria, with an average length of residence in the US of approximately 5 years. The mean respondent age was 36.8 years. The majority of the participants spoke English very well/well (87.5%) and had a personal healthcare provider (71%). The themes and subthemes emerging from participant interviews are discussed below and in a summary table presented in Table 2.

### 3.1. Women’s Overall Experiences Participating in the Cervical Health Intervention

#### 3.1.1. Empowerment and Connections

The interview process began by asking the women what their initial experience was participating in the cervical health intervention. Nearly all the women acknowledged that they were excited to participate and had a positive experience. For example, a woman responded, “Okay, overall, it was a good experience.”, and another woman said, “I think it was great.” Following the intervention, participants felt empowered to take the initiative for their own cervical health. Women understood the importance of being proactive, and one woman noted, “it’s really, you know, helps me to be well informed about taking, a very quick or prompt step about making sure that things that are meant to be prevented are prevented.”

Women were joyous that the intervention was offered to diverse women; one stated that she “liked the fact that it was made available, especially to women of color.” A few of the women commented that they were joyous when they joined the Zoom because they were surrounded by other Black and African American women who were present to gain new information just as they were. One of the participants expressed, “I had never had such performed, and then doing the video conference during the Zoom session with the other participants…it was very enlightening to know that I was not alone.” Women felt connected, seeing a cohort of researchers and participants that shared their ethnicity and values. A woman noted “after I participated in the educational section, it was, the discussions that we had with everyone present, I learned a lot about HPV, and the tests, and all of that, I think, was really helpful.”

#### 3.1.2. I Feel Enlightened

Overall, participants were satisfied with the information provided in the intervention, and they felt it was educational. A few women commented that they learned a lot about HPV and cervical health. One woman said, “there was definitely an increase in my … in the knowledge about HPV for me.” Other responses suggested that some women felt enlightened. For example, a woman noted, “And for me, it was like, an eye opener, I even learned that males can also have HPV, and that’s something I didn’t know before.” Building upon HPV and cervical cancer education provided participants with the information and tools they needed to make good, informed choices with their cervical health. One participant stated, “I was exposed to a lot of information, a lot of depth that I was not aware of. It has really helped my thinking and also the way I perceive my health.”

#### 3.1.3. Accessible and Engaging

We wanted to ensure that our presentation was accessible to women of all literacy and education levels. Most participants were engaged by the cohesiveness of the presentation. When asked if the presentation was difficult to understand, a participant responded, “No, I didn’t feel that there were too many medical jargons. When necessary, you (the presenter) took the time to break it down into layman’s terms and paused to add clarity.” The presenter successfully conveyed the importance of HPV and cervical cancer screening, and they answered any questions the participants had. This was well received by the participants. Another woman said, “I think that what you are doing is just versatile. You are addressing a lot of issues, and you are cutting across every sphere…for people that are fearful, for people who are well exposed, for people that don’t seem to have an understanding.”

Other women felt that some medical terms would be incomprehensible for women of lower literacy levels. For example, one woman exclaimed, “Maybe, you know, using all those medical terms, they wouldn’t understand…Because there were just some things that I did not, I mean… Well like that I did not know.” Another woman had a similar sentiment. She protested that “Kind of the medical jargon… yeah…It was too big. Because there were just some things that I did not, I mean… Well like that I did not know.” These participants agreed that, even though the terms were defined, immigrant women and women who are less literate may be less engaged.

### 3.2. Participant Experiences Performing HPV Self-Collection

#### 3.2.1. Not as Difficult as I Thought

Several women felt that collecting the sample was a seamless process. The instructions provided with the kit and covered in the educational session allowed most of the women to feel confident performing the process at home. Many participants had never used a self-collection kit, but a woman explained, “when I eventually did it, it was so so so painless.” After collecting the sample, the participants were confident that anyone can self-sample. For example, one woman said, “It’s not something scary, it’s not something that is not achievable by anybody.” One woman equated the process to something that is more familiar, she explained, “I’m comfortable using tampons. So, it wasn’t a big deal for me to just self-administer the test.” Due to the simplicity of sampling at home, most participants preferred the option of HPV self-sampling to clinician-based tests.

#### 3.2.2. Convenience

We found that the convenience of self-sampling alleviated a few of our participants’ barriers. The inconvenience of clinician-based testing restricts many women from being able to maintain routine cervical cancer screenings. A woman emphasized, “I have a baby at hand, and it would have been inconveniencing for me to like, go with him or find somewhere to keep him.” Women benefited from the fact that HPV self-sampling required no scheduling changes. One participant stated, “I was also happy to like get a kit and do it from the convenience of my house, not needing to go out to schedule an appointment to get that done.”

#### 3.2.3. Fear

We encountered some participants who had perceived fears of performing the HPV self-sample kit. These women chose not to use the kit. Participants either had negative experiences previously with clinician-based testing, were hesitant about inserting a vaginal swab, or preferred to see their doctor. A woman shared with us a previous experience she had, causing her to be fearful of participating. She explained, “I didn’t realize that, I have started dreading invasive procedures.” Another participant admitted, “I know I need it as much as possible. Maybe I have a little bit of fear.” In contrast, a woman preferred clinician-based testing, and she expressed, “at first, I was scared I wouldn’t be able to do it properly. I didn’t want complications… so I prefer that I should see my doctor.”

### 3.3. Suggestions to Improve Intervention Implementation

#### 3.3.1. Communication Preference

With regard to communication between the participants and the researchers, many of the respondents expressed a preference for emails and or text messages. A participant explained, “I don’t mind email or message… But email or text message will be just fine for me.” Similarly, a woman stated, “For me I think messages and emails worked for me.” Many of the women who participated in the follow-up interview discussed that they were comfortable with emails and that they check their emails on a regular basis. A woman expressed, “Email, I think email is the least invasive.” Although this was a common consensus, responses favoring emails and text-messages were representative of women who currently use the communication modes.

However, other participants noted that “phone calls would be more personal because a lot of people might not check their emails.” Many people do not regularly check their primary email accounts or even text messages unless they are expecting something important to be sent. The women recommended that social media is also an effective approach to reach a more diverse audience. A participant recommended, “I think they should improve in their social media.” Many people are now connected via social media platforms and other multimedia applications. Another participant noted, “many people are now used to the internet. So, I think that if you want to reach them you should use the internet.”

#### 3.3.2. Platform for Implementation

An aspect that was discussed by multiple participants was the lack of a platform or repository to return to if they wanted to listen to the presentation again. Women were provided with an electronic PowerPoint and the choice to receive a printed version of the material. It was suggested that, if we desired to reach more African American and African-born Black women, “having a website or even a social media page, where you can post info graphs and key information so people could quickly scroll through, would be more helpful.” Increased flexibility of the education session was a common suggestion for improvement. A respondent recommended that since “flexibility is a big deal for most people…why don’t you create a web page and record the session.” Women explained that they liked the information presented throughout the course of the study, but it had a lot of depth. They would like it if they could have a format available to guide them through the details presented in the study on their own time. This would allow them to access the contents of the PowerPoint and be able to ask the research group questions via an online format if they had any uncertainties.

#### 3.3.3. Intervention to Be Delivered in Other Languages

Language was another major concern amongst participants. A woman shared concern for how we were seeking to recruit African-born Black women, but only offered the intervention in English. To recruit more African born women, a participant recommended, “If there’s a way even for future intervention, if they’re still targeting that kind of population, a way to make it work, get someone who can do translation.” Another participant emphasized that “There’s potential that the messages could be tailored more to the demographics of the people…” Offering the intervention in languages such as Swahili and French was a common suggestion for improvement.

A couple of women lacked the opportunity to engage in the study due to language barriers. One woman stated, “I had, I knew people that I would have loved to benefit from the intervention, but due to the language and communication barrier, they were not able to participate.”

#### 3.3.4. Spousal Involvement

Relationships such as marriage are known to greatly influence one’s health-seeking behavior. We received varying responses about whether it would be beneficial to have one’s spouse participate in the intervention or not. Some women were in support of spousal involvement. When asked if the partner was involved, a woman shared, “he was aware and is in support.” Another woman received a lot of support. She commented, “Yeah, he was happy that I was doing it. I think he’s also interested in like women’s health, so he was happy.” Certain religious, financial, or educational reasons may require a woman’s spouse to participate in the intervention. A participant emphasized, “maybe for not well-educated, not financially stable…and some ethnic groups that depend on their husbands to be able to give information about them…one might need to get the partner involved.”

Other women were comfortable participating in the intervention and performing the sample without the support of their spouse. One participant felt “it isn’t necessary. It’s like a regular screening. It is one of the steps that I need to take for myself to keep in good health.” Likewise, a woman stated, “I don’t think it’s necessary. I’m married but my husband, you know, didn’t help me with anything. I think it’s something that you can do on your own…” However, women agreed that both partners should be involved in the education session. A participant noted, “But it’s always important for them to know about like, sexual health and education…there might be a need for more tailoring to include men in the messages.”

## 4. Discussion

This study contextualizes the experiences of African-born Black and African American women who participated in Health is Wealth: a cervical health intervention. This intervention combines an HPV education session with a complimentary self-sampling kit, which were both well received by participants. In this study, women expressed their likes, dislikes, concerns, recommendations, and preferences related to the HPV education session and the self-sampling kit. The themes and subthemes arising from the data have implications for future interventions. Numerous findings emerged from the interviews that suggest community-based interventions, such as Health is Wealth: a cervical health intervention, have the potential to decrease disparities in cervical cancer screening rates amongst African American and African-born Black women.

We identified facilitators to successfully increase the uptake of our cervical health intervention. Minority women are highly under-represented in clinical research despite their over-representation in both negative health outcomes and disease [21]. Often, African American and African-born Black women are not offered the opportunity to participate in clinical research or health-based interventions, as expressed by participants from our study [22]. Women valued and felt connected by the culturally competent environment as they were being guided toward achieving more control over their cervical health. Research shows that perceived social support has a positive effect on cervical cancer screening uptake among women, whether it be from including one’s spouse or if interventions involve community health workers and peers from their community [23,24]. Providing cervical cancer education, while addressing unique issues among these populations, invoked positive attitudes among participants because they were given resources and information that fostered an awareness of the importance of routine cervical cancer screening, ultimately empowering them to take the initiative for their own cervical health.

All the women confirmed that their deficiencies in HPV and cervical cancer health literacy were addressed in an acceptable manner. An ideal duration of a health-education intervention depends on the targeted groups and the goal of the intervention. Research aimed at identifying features that may increase participation in health-education interventions has observed that respondents prefer short and convenient sessions. Most individuals are willing to participate in one intervention lasting 30–60 min, either in a group or one-on-one [25]. Cervical cancer and HPV education should be an essential component of interventions aimed at increasing screening rates among minoritized ethnic groups, such as African American and African-born Black women. Increasing women’s cervical cancer literacy increased their awareness of HPV screening guidelines and cervical cancer disparities amongst African American and African-born Black women. After becoming more educated, participants were interested in becoming more proactive with their own screening routines. This finding is congruent with previous research demonstrating that educational interventions increase cervical cancer screening uptake rates amongst diverse and high-risk populations [12,26].

We discovered a consensus that different communication approaches have the potential to reach a more diverse audience for participation and retention. Participants identified phone calls, emails, and text messages as being preferable forms of communication throughout the intervention because they use these communication modes frequently. However, we received recommendations to communicate through social media platforms to reach a more diverse audience. The current literature supports that interventions should often leverage online social networks for communication, as many people use them to seek information about health, parenting, and a variety of other topics [27]. More research is needed to directly explore how social media can increase cervical cancer screening rates among African American and African-born Black women, but evidence exists supporting it as an effective way to positively increase awareness of HPV health information to targeted populations [28]. As more African American and African-born Black women become aware of the importance of HPV and cervical cancer screening, they are more likely to routinely screen for cervical cancer. Evidence suggests that social media may improve cancer screening and early diagnosis, as the current literature maps evaluation frameworks to measure behavioral change [29].

Another key observation was that flexibility was an important factor contributing to participation in the study. Participants recommended the intervention be offered in a multicomponent format, having both a Zoom session and an interactive webpage to broaden the reach to more African American and African-born Black communities. There were some challenges with recruitment involving scheduling a cohesive time for participants to meet in Zoom sessions. Ultimately, this restricted our sample size. Implementing multicomponent research interventions combines multiple strategies to engage participants, leading to broader and more effective outcomes [30]. Research aiming to increase intervention participation by individuals from under-represented groups supports a multicomponent intervention approach as a way to increase diversity among research participants [31]. The development of a multicomponent platform has the capacity to target multiple barriers that African American and African-born Black women face to HPV and cervical cancer screening, given its capacity to target multiple specific needs of a community and engage more diverse groups.

Development of interventions that include women and their spouses could improve cervical cancer screening rates among African American and African-born Black women. Ethnic women who have more spousal support are more likely to have positive attitudes toward screening and have fewer perceived barriers [32,33]. Marriage is associated with earlier diagnosis and a favorable prognosis among US women, even after adjusting for insurance status [34]. Prompted discussions should focus on properly informing men on the transmission of HPV infection as well as the development of other types of HPV-related cancers to emphasize the role of healthy sexual behavior in the prevention of HPV transmission.

From the interviews, we were able to collect opportunities to improve uptake by African American and African-born Black women. For future iterations of this research, a bilingual African-born Black woman fluent in French or Swahili, or a certified translator or interpreter, will be trained to deliver the intervention in French or Swahili for African-born Black. Sub-Saharan Africa is linguistically diverse. A language variety should be incorporated into future interventions to provide women who prefer or only speak native languages to participate.

In this current study, participant recruitment was conducted by trained African American and African-born Black women from the research team. The intervention was delivered by African American and African-born Black women for ethnic congruence. The literature on the effect of race and ethnicity on outcomes of healthcare emphasizes that race-concordant patient–provider relationships are effective in reducing racial and ethnic disparities [35]. Our research observed that African American and African-born Black women valued, or even preferred, that the intervention be delivered by a same-race health advisor. Respondents felt more confident and empowered over their own cervical health, encouraged by researchers who shared their same racial or ethnic background.

### Limitations

While this study provides valuable insights into the experiences of African American and African-born Black women with a cervical health intervention, several limitations must be acknowledged. First, the relatively small sample size (n = 24) limits the generalizability of findings to the broader population of African American and African-born Black women in the U.S. However, data saturation was achieved despite the small, region-specific sample. The data may not reflect the vast diversity of experiences obtainable across different geographic or cultural contexts within the US. Second, most women in this sample had higher educational attainment, which could govern their health literacy, willingness to participate, and optimism toward self-collection. Third, women with limited internet access or proficiency in technology were also potentially excluded from participation by hosting the education session via Zoom. Future interventions should aim to maximize accessibility by exploring hybrid models. Lastly, the intervention was only offered in English, which, consequently, omitted participation by non-English-speaking women.

## 5. Conclusions

Interventions that address the needs of a target population can improve participants’ acceptability and engagement with the intervention, leading to improved outcomes. Interventions like Health is Wealth: a cervical health intervention, which offers education and the provision of HPV self-sampling kits, can empower African American and African-born Black women to participate in preventative cervical cancer screening and follow-up care. By understanding their unique experiences and perspectives, we can develop strategies to promote screening amongst a population with a high burden of disease. We have several opportunities to improve the intervention and scale it to larger audiences. Offering the intervention in major African languages gives the opportunity to reach African-born Black women who may prefer the intervention in their native language. The results and conclusions made from this study will be used to refine the intervention for future implementation. Interventions such as Health is Wealth: a cervical health intervention provide a framework to healthcare providers and researchers for promoting cancer screening and reaching medically underserved women with preventive care.

## Figures and Tables

**Table 1:** Participants’ characteristics (24).

Variable	Category	Mean or Frequency	Percentage
Age (years)		36.8	
Marital status	Married or partnered	16	66.7
	Separated/divorced	2	8.3
	Single	6	25
Educational level	High school or GED	1	4.2
	2 years of college	3	12.5
	College	11	45.8
	Master’s degree or higher	9	37.5
Household income	Less than $15,000	1	4.2
	$15,000–$34,999	10	41.7
	$35,000–≤$49,999	3	12.5
	$50,000–≤$79,999	6	25
	≥$80,000	4	16.7
Has Health Insurance	No	3	14.3
	Yes	21	87.5
Race/Ethnicity	African American	10	41.7
	African Born Black	14	58.3

**Table 2:** Themes and subthemes emerging from participants’ interviews.

Themes	Subthemes
Women’s overall experiences participating in the Cervical Health Intervention	Empowerment and connections I feel enlightened Accessible and engaging
Participant experiences performing HPV self-collection	Not as difficult as I thought Convenience Fear
Suggestions to improve intervention implementation	Communication preference Platform for implementation Intervention in other languages Spousal involvement

## Data Availability

The data that support the findings of this study are available from the corresponding author upon reasonable request.

## Supplementary Materials

The following supporting information can be downloaded at: https://www.mdpi.com/article/10.3390/healthcare13192389/s1, Post-Intervention Interview Questions.
