# Supplementary material for: African American and African-Born Black Women’s Perspectives and Experiences with a Cervical Health Education and HPV-Self Sampling Intervention
Source: Healthcare (Basel). 2025 Sep 23;13(19):2389. doi: 10.3390/healthcare13192389 (PMC12523894; doi:10.3390/healthcare13192389)
Supplement: Supplementary file 1 [file healthcare-13-02389-s001.zip › healthcare-3699059-supplementary.pdf]

African American and African Born-Black Women's Perspectives and Experiences with a Cervical Health Education and HPV-Self Sampling Intervention.

Post-Intervention Interview Questions

Thank you for choosing to participate in this interview! This interview should take between 15-30 minutes and the purpose of this interview is to collect feedback about your experience participating in this research study and collect suggestions for improvement. Your responses are highly appreciated and valued.

1. Please talk briefly about your experience participating in the Health is Wealth Intervention.
    a. What did you like or dislike about the intervention?
    b. What was easy and what was challenging about it?
    c. Based on your experience can you discuss any concerns you have about the intervention comprehension.
    d. What changes would you suggest to improve the delivery and acceptability of the intervention.
    e. What changes would you suggest in communication with participants for this intervention- text messages, email, letters, phone calls??
    f. What additional information do you think we should include in the intervention.
2. If you recall, you received an HPV kit,
    a. What have you done with the provided kit?
    b. If you have used the kit, what was your experience with that?
        i. What challenges did you face with collection, mailing, and receiving your results?
        ii. How satisfied were you with the process? Why?
        iii. How would you describe your husband/partner support for completing the HPV self-sampling? Do you think partners should have been part of the intervention session
    c. If you have not used the kit, why did you choose not to complete the HPV self-sampling?
    d. What have you done with the kit?
    e. What do you plan to do with the kit?
3. What suggestions do you have for the research team for us to use to make this intervention better for other women?
4. What suggestions do you have for the research team to use to scale up this intervention to make it more available to more women?
5. Do you have any other feedback, questions, concerns or comments about your experience participating in this intervention?

Thank you for your time!
